# Integrating Sperm Microscopy, Environmental Exposures, and Lifestyle Factors for Male Fertility Analysis: Protocol for the Nippon Semen and Environmental Exposure Database (N-SEED) Cross-Sectional Study

**DOI:** 10.2196/93803

**Published:** 2026-04-17

**Authors:** Tomoko Oguri, Kosuke Kojo, Yoshiaki Endo, Takaaki Matsuda, Haruhiko Midorikawa, Ayumi Nakazono, Kaoru Yanagida, Atsushi Ikeda, Hiroyuki Nishiyama, Isamu Ogura

**Affiliations:** 1 Research Institute of Science for Safety and Sustainability National Institute of Advanced Industrial Science and Technology Tsukuba, Ibaraki Japan; 2 Department of Urology Institute of Medicine University of Tsukuba Tsukuba, Ibaraki Japan; 3 Tsukuba Clinical Research & Development Organization University of Tsukuba Tsukuba, Ibaraki Japan; 4 Center for Human Reproduction International University of Health and Welfare Hospital Nasushiobara, Tochigi Japan; 5 Department of Physical Therapy School of Health Science International University of Health and Welfare Otawara, Tochigi Japan; 6 Department of Endocrinology and Metabolism University of Tsukuba Hospital Tsukuba, Ibaraki Japan; 7 Department of Psychiatry University of Tsukuba Hospital Tsukuba, Ibaraki Japan

**Keywords:** computer-assisted image analysis, endocrine disruptors, environmental exposure, erectile dysfunction, healthy volunteers, male infertility, oxidative stress, sleep, sperm motility, testosterone

## Abstract

**Background:**

Conventional semen analysis does not fully capture male reproductive potential. The sperm DNA fragmentation index (DFI) may detect latent infertility, although the sperm chromatin structure assay (SCSA) is costly and technically demanding. Image-based analysis of semen microscopy, including artificial intelligence (AI), may enable lower-cost noninvasive assessment. However, progress is limited by a lack of standardized multimodal datasets linking sperm images with the DFI and relevant covariates.

**Objective:**

This protocol describes the initial phase of the Nippon Semen and Environmental Exposure Database (N-SEED) initiative. The study aims to (1) establish standardized acquisition and quality control procedures for sperm microscopy/video and DFI measurement; (2) evaluate the feasibility and magnitude of associations between predefined image-/video-derived variables and the DFI; and (3) characterize clinical, environmental, physical, and lifestyle factors that may act as candidate correlates or confounders for subsequent image-based fertility research.

**Methods:**

This multi-institutional cross-sectional study centralized clinical assessment and specimen collection at a single site in Japan. The prespecified group-specific enrollment targets were 25 for group 1 (a relatively homogeneous low-risk cohort for DFI quality control) and 100 for group 2 (apparently healthy community-based adult volunteers for exploratory association analyses). Microscopic sperm videos, automated semen parameters, and sperm oxidative stress data were obtained at collection. SCSA-based DFI assays are currently being performed sequentially. Group 2 participants additionally provided fasting blood and early-morning urine samples for endocrine, metabolic, environmental chemical, and elemental assays. They also underwent physical measurements and completed structured questionnaires/interviews on lifestyle, nutrition, sleep, psychological status, sexual function, and collection conditions. The primary outcome measure is the continuous DFI assessed with SCSA. Group 1 data will be used to evaluate intraday, interday, and interfacility variability and to fix flow cytometry gating settings. In groups 1 and 2, predefined image-/video-derived summary variables after standardized preprocessing will be analyzed against the continuous DFI using correlation analyses and simple linear regression to assess whether sperm microscopy data contain information relevant to sperm DNA integrity. Exploratory categorical DFI analyses will use simple logistic regression. Secondary exploratory analyses will examine candidate covariates and data completeness, including sensitivity analyses.

**Results:**

The study was registered in the University Hospital Medical Information Network Clinical Trials Registry on January 18, 2026. Between January 18 and February 21, 2026, 124 unique participants were recruited (group 1: n=25; group 2: n=103; both groups: n=4). Sample and data collection were completed on February 21, 2026. Biospecimen assays and primary association analyses are expected to be completed by March 2028.

**Conclusions:**

This initial N-SEED phase will deliver a standardized, quality-controlled multimodal resource linking sperm microscopy/video data with the DFI and broader physiological and environmental measures. Its immediate contribution is not to provide an immediately deployable clinical AI tool but rather to support feasibility assessment, confounder identification, and planning for subsequent database expansion and external validation of image-based male fertility assessment methods.

**Trial Registration:**

UMIN Clinical Trials Registry UMIN000060395; https://tinyurl.com/mryfymw5

**International Registered Report Identifier (IRRID):**

DERR1-10.2196/93803

## Introduction

### Background

Concern about declining semen quality has grown because meta-analytic studies have suggested substantial reductions in sperm concentration and total sperm count in some populations over recent decades [1,2]. At the same time, temporal inferences remain sensitive to differences in population selection, laboratory methodology, geography, and study design [3,4]. Thus, although adverse trends in semen quality represent an important public health concern, their magnitude and generalizability remain debated. Several interacting explanations have been proposed, including aging and genetic susceptibility, as well as potentially modifiable factors, such as obesity, smoking, alcohol use, psychological stress, reduced physical activity, heat exposure, and environmental chemical exposures, particularly to endocrine-disrupting compounds [5,6]. The public health relevance extends beyond delayed conception alone, because impaired semen quality may also correlate with future cardiometabolic disease, malignancy, hospitalization, and reduced longevity [7-9].

However, population-level concerns about semen quality trends should be distinguished from the clinical problem of assessing fertility potential in individual men. Low sperm concentrations or other abnormal semen parameters increase the probability of subfertility at the population level, but except for severe phenotypes, such as azoospermia, conventional semen analysis alone does not fully capture individual reproductive potential [10,11]. This limitation has increased interest in sperm DNA fragmentation (SDF), a form of sperm genomic damage, and in the DNA fragmentation index (DFI), a quantitative indicator of sperm chromatin integrity. The DFI is commonly defined as the proportion of sperm with fragmented or acid-denaturable DNA [12,13]. It is typically quantified using the sperm chromatin structure assay (SCSA), which involves acid-induced DNA denaturation, acridine orange staining, and flow cytometry [14,15]. A higher DFI has been associated with prolonged time to pregnancy and less favorable assisted reproductive technology outcomes in some settings, although thresholds and clinical indications remain context dependent [15-17].

Despite its potential clinical relevance, SCSA-based DFI assessment is more technically demanding than routine semen analysis and has limited scalability for broader screening or repeated measurement [10,13,15]. This creates interest in lower-cost, noninvasive approaches that could extract information relevant to sperm DNA integrity from standard semen microscopy.

### Prior Work

Recent artificial intelligence (AI) and machine learning (ML) applications in andrology have mainly focused on relatively narrow input domains, such as sperm images/videos, semen parameters, or single-modality clinical variables. Reviews consistently emphasize the need for larger standardized datasets, multimodal integration, and external validation before clinical adoption [18-20]. Progress is being made in attempts to predict the DFI using deep learning based on microscopic semen images and video data obtained through relatively inexpensive and noninvasive examination [21,22]. However, practical AI development remains constrained by the lack of high-quality datasets that directly link standardized sperm microscopy with the DFI.

The functional quality of sperm is influenced not only by testicular factors but also by multidimensional behavioral, physiological, and environmental influences. These include the abstinence period [23]; the ejaculation frequency [24]; collection-context factors, such as the duration of sexual arousal [25] and intensity of orgasm [26]; environmental chemical exposures [27,28]; and lifestyle habits [29]. Accordingly, the broader physiological and environmental context has been underrepresented in many existing AI-oriented datasets and study designs.

Attempts have also been made to predict male fertility using AI analysis of large-scale databases of blood endocrine profiles related to testicular function, independent of semen findings [30]. Although such endogenous physiological factors may be informative, many existing large-scale datasets rely on retrospective observations in patients with infertility, where diverse pathologies limit adjustment for confounding. In contrast, prospective data collection in non-clinic-based community populations may reduce the influence of disease factors that directly impair fertility. However, clinical studies involving men are relatively rare [31], and when semen analysis is included, volunteer bias can become substantial [32]. Consequently, defining truly healthy reference values and exploring candidate risk factors remain difficult.

ML has been used to predict semen findings from lifestyle habits [33-35]. Similarly, AI models have been developed to automatically analyze semen parameters from sperm images [36-38]. In parallel, systematic reviews on the effects of environmental chemicals and air pollution on semen findings have been published in recent years [39,40]. However, to the best of our knowledge, no dataset integrating sperm images with comprehensive environmental, physiological, and lifestyle data to evaluate association with the DFI, and to support future predictive modeling, in a non-clinic-based general population where the influence of overt disease factors is minimized has been established.

### Study Objectives

This protocol paper describes the initial phase of the Nippon Semen and Environmental Exposure Database (N-SEED) initiative as a multicenter cross-sectional study. N-SEED is designed as a long-term, scalable database infrastructure and is not intended to be completed in a single year. As a preliminary step, the 2026 project prospectively collected biospecimens (urine, blood, and semen) and related data (semen analysis, physical measurements, and questionnaire/interview data) from apparently healthy community-based adult men. These data will be used to statistically evaluate the associations between sperm microscopy image/video data and sperm functional assessment measures, including the DFI. The ultimate long-term goal is to support the development and validation of image-based male fertility assessment technologies. This initial phase focuses on data collection feasibility, measurement quality control, and analytical readiness. Accordingly, this initial phase is not intended to develop or externally validate a clinically deployable AI model. Rather, it is intended to establish standardized acquisition and quality control procedures; estimate the magnitude and variability of image-DFI associations; identify candidate confounders and features; and inform formal sample-size planning, model development, and independent validation strategies for later phases. Specifically, we will conduct a preliminary assessment focusing on three key aspects:

- First, we will evaluate interfacility variability and perform intrafacility quality control of the DFI testing method. In group 1, which is designed primarily for quality control of DFI testing, we will assess the reliability of the measurement system.
- Second, we will prospectively collect standardized microscopic sperm videos and related multimodal data. Initial association analyses between sperm image/video data and the DFI will then be conducted to evaluate feasibility and inform subsequent study design (groups 1 and 2).
- Finally, we will explore multilayered factors potentially related to the DFI and sperm image/video data, including standard semen analysis parameters, endocrine and metabolic biomarkers, environmental chemicals, physical measurements, and lifestyle factors, to support confounding adjustment and hypothesis generation in future phases (group 2).

## Methods

### Study Design

The study has been registered in the University Hospital Medical Information Network Clinical Trials Registry (UMIN-CTR; #UMIN000060395; registration date: January 18, 2026; disclosure date: January 19, 2026).

This is a multi-institutional cross-sectional study with clinical data collection centralized at a single site to ensure high-quality standardization. One of the aims is to construct a foundational database capable of supporting future methodological development (including potential AI approaches), external validation, and generation of new hypotheses. To this end, we prospectively and systematically collected multimodal information (semen analysis, physical measurements, and questionnaires) centered on microscopic sperm image/video data obtained from apparently healthy community-based adult men, together with biological samples (semen, blood, and urine).

A characteristic of this study is the use of two participant groups with different inclusion criteria according to the analytic purpose. Participants in group 1 were recruited to ensure the reproducibility and stability of the DFI testing method. By minimizing confounding factors that may adversely affect sperm quality, such as aging, obesity, underweight, and smoking, and by limiting the group to men with a high ejaculation frequency, we aimed to identify a population expected to maintain high spermatogenic function. In this group, only semen was collected as a biological sample. In contrast, participants in group 2 were included to ensure broader representation of potential risk factors associated with a worsening DFI in otherwise apparently healthy community-based volunteers. In this group, in addition to semen, blood and urine samples were collected to examine the effects of nutritional and endocrine profiles and environmental exposure.

### Study Setting

The National Institute of Advanced Industrial Science and Technology (AIST, Tsukuba, Japan) serves as the central hub for data management and analysis in this study. AIST is responsible for planning research, obtaining ethics committee approval, receiving and storing samples and data, designing and constructing the database, overseeing clinical testing and chemical analysis (including external outsourcing), and coordinating statistical analyses and subsequent methodological development as the database expands.

Research tasks, such as reviewing the research plan, recruiting participants, obtaining informed consent at the visit, collecting interview and questionnaire data and physical measurements, collecting biological samples, acquiring general semen analysis parameters (including sperm concentration and motility), and acquiring sperm microscopic videos, were conducted at the Osaka Pharmacology Clinical Research Hospital (Osaka, Japan). This hospital conducts clinical research as a single third-party institution following standardized procedures. In addition, expert supervision in reproductive medicine, endocrinology, psychiatry, analytical chemistry, physiotherapy, epidemiology, and hygiene, as well as specific measurement items (eg, environmental chemical measurements), is conducted in collaboration with multiple universities and research institutions.

The recruitment and registration period ran from January 18 to February 21, 2026. Data and biological sample collection was completed during visits in this period. No follow-up is planned because of the cross-sectional design. Biological sample analyses will be performed sequentially after collection and are scheduled to continue until March 2028, which marks the end of the study period.

### Participants

#### Eligibility Criteria

The inclusion criteria for the participants are presented in Table 1. In group 1, the eligibility criteria were designed to minimize factors adversely affecting spermatogenic function for quality control of DFI measurement values. Specifically, group 1 included only men who were sexually active young adults (18-24 years old), had a high frequency of ejaculation (average of ≥10 per month), had a standard body type (BMI 18.5 to <25 kg/m²), and had never smoked. Group 2 had broader inclusion criteria to explore DFI risk factors in men from diverse backgrounds. The target population included men of general reproductive age (18-41 years) with minimal ejaculatory function (average of ≥1 time per month). However, to ensure that the blood and urine test results were accurate, compliance with visits during the designated time (8:00 to 11:00 A.M.), fasting the previous night, and a two-visit schedule was mandatory for this group.

**Table 1:** Inclusion criteria for groups 1 and 2.

Criteria	Group 1 (quality control)	Group 2 (exploratory)
Age (years)	18 to <25 years	18 to <42 years
BMI (kg/m²)	18.5 to <25.0	No specific restriction
Ejaculation frequency (times/month; average over the past 6 months)	≥10	≥1
Smoking status	Never smoked [41]^a^	No specific restriction
Medical history	No history of congenital anomalies, male reproductive organ diseases/trauma, varicocele, malignant tumors, endocrine disorders, or autoimmune diseases	Same as group 1
Medication	No history of hormone medication use [42-45]^b^	Same as group 1
Study compliance	Able to comply with 48-144 h of abstinence	Able to comply with 48-144 h of abstinence, visit between 8:00 and 11:00 A.M. (fasting condition), and attend two visits

^a^Lifetime consumption<100 cigarettes, no smoking in the past 6 months, and no smoking during the study period [41].

^b^Hormone medication and other drug exclusion criteria: participants who had used thyroid hormone preparations [42] or 5α-reductase inhibitors (eg, finasteride, dutasteride) [43] within the past 6 months were excluded. Minoxidil use was permitted [44]. Corticosteroids were generally permitted, except when used for self-reported autoimmune diseases [45]; however, topical or inhaled corticosteroids used for conditions such as atopic dermatitis or asthma were acceptable. Other medications were not exclusionary; current medication use (including that of drugs that may affect ejaculation or sexual function) was collected in detail via questionnaires/interviews and will be considered in sensitivity analyses, as appropriate.

The exclusion criteria were identical for both groups. We excluded individuals with a history of congenital anomalies or diseases of the male reproductive organs, varicocele, malignant tumors, endocrine diseases, or a history of hormone medication use. Additionally, individuals who might experience unjust disadvantages from participating, those who had difficulty providing consent because of cognitive impairment, or those judged by the study physician to be in poor physical condition on the day of the survey (eg., acute illness symptoms) were considered ineligible. If a participant in group 1 also met the requirements of group 2 and wished to participate, co-enrollment in both groups was permitted. Such cases were identifiable through study registration records. Because groups 1 and 2 were established for different purposes rather than as formal independent comparison arms, co-enrolled records were retained as group-specific records but were not treated as statistically independent in any cross-group descriptive comparison or pooled participant-level analysis.

#### Source Population and Recruitment

To reduce clinic-based sampling bias (ie, overrepresentation of men seeking fertility evaluation), we recruited men residing in Japan who were registered in a volunteer panel managed by InCROM Co, Ltd (Nishiwaki, Japan) rather than through infertility clinics. After eligibility confirmation, participants were referred to the Osaka Pharmacology Clinical Research Hospital, a clinical research implementation facility, where written informed consent was obtained. This study concerns semen-based measures of biological male reproductive function; gender identity and sexual orientation were not assessed, and sex/gender was not analyzed as a separate covariate in this initial phase. Given that study participation required morning fasting visits and (for group 2) two visits, we anticipated potential overrepresentation of individuals with greater scheduling flexibility. To characterize this quantitatively, age and recruitment-related sociodemographic variables (eg, employment status, shift work, education, and income) were systematically collected. Their distributions, including “prefer not to answer” responses where applicable, will be reported.

#### Sample Size

Considering biological sample processing, assay capacity, and the budget in this initial phase, the target group-specific enrollment was set at 25 for group 1 and 100 for group 2. Because co-enrollment was permitted, these targets refer to group-specific enrollment records; therefore, the total number of unique individuals across both groups may be smaller than the sum of the two group-specific counts. The two groups address different objectives. Group 1 is intended for quality control and descriptive characterization of the DFI measurement system in a relatively homogeneous low-risk population, rather than formal hypothesis testing. Group 2 is intended for feasibility-oriented initial association analyses between sperm image/video data and the DFI and exploratory analyses of candidate factors. Therefore, the sample size was determined pragmatically rather than via a formal power calculation for a specific effect size. We acknowledge that this initial phase may be underpowered to detect small associations or to support multivariable models with many predictors. Accordingly, effect estimates, CIs, and consistency of direction will be emphasized, and the findings will be used to inform variable prioritization and formal sample size planning for subsequent phases.

### Variables and Data Collection

A comprehensive dataset was collected to construct a multimodal database linking sperm microscopy image/video data with semen examination results, the DFI, and extensive clinical, environmental, and lifestyle covariates. The primary purpose of this initial phase is to establish standardized data collection and enable association analyses and hypothesis generation. The dataset will also provide a foundation for subsequent phases, including stepwise development and external validation of image-based analytical approaches (including AI) as the database expands.

Most participant-reported data (baseline characteristics, lifestyle factors, psychological status, sexual function, and collection-related subjective assessments) were obtained using paper-based structured self-administered questionnaires in Japanese. Study staff checked completeness with each participant at the visit. Physician-conducted interviews were limited to confirmation of eligibility (including poor physical condition on the day of the visit) and clarification of clinically complex medical history, reproductive/urological history, infertility treatment history, and medication use that may have been difficult to classify accurately through self-report alone. These interviews followed a prespecified oral checklist aligned with the eligibility criteria. For sensitive socioeconomic and sexual/reproductive items, participants could choose “prefer not to answer.”

#### Baseline Characteristics and Environmental Exposures

Data on baseline characteristics were collected through these structured self-administered questionnaires and through physician-conducted interviews for clinically complex eligibility items. Demographic and socioeconomic factors included age, height, weight, educational attainment, marital status, and household income [46]. Regarding medical background, current and past medical histories were obtained. Regarding current medications, we verified the use of treatments for androgenetic alopecia, drugs for erectile dysfunction, and zinc supplementation. Furthermore, as part of the reproductive background, the partner’s pregnancy history, history of infertility treatment [47,48], the participants’ desire for children [49], and experience using home sperm-testing kits [50] were investigated.

Regarding environmental and occupational exposures, employment status, including shift work, was assessed [51]. Work in high-temperature environments and the frequency of laptop use on the lap were also assessed to evaluate potential thermal effects on spermatogenesis [52].

#### Lifestyle Factors, Psychological Status, and Sexual Function

Lifestyle factors, psychological status, and sexual function were evaluated multidimensionally using structured self-administered questionnaires, incorporating validated scales and specific self-developed questions. Where available, validated Japanese versions or established Japanese translations of standard instruments were used. Regarding lifestyle habits, data on smoking habits (Brinkman index; daily number of cigarettes × years) [53], alcohol consumption (Alcohol Use Disorders Identification Test–Consumption [AUDIT-C]) [54], and sauna/bathing habits (self-developed questionnaire) were collected. Nutritional intake was assessed using the Brief-type Self-Administered Diet History Questionnaire (BDHQ) [55]. Sleep quality was evaluated using the Japanese version of the Pittsburgh Sleep Quality Index (PSQI-J) [56], chronotype using the reduced Morningness-Eveningness Questionnaire (rMEQ) [57], and physical activity levels using the International Physical Activity Questionnaire (IPAQ) [58].

Psychological status was assessed using the Perceived Stress Scale (PSS) [59] and the Kessler Psychological Distress Scale (K6) [60]. Sexual function and urological symptoms were comprehensively evaluated using the Sexual Health Inventory for Men (SHIM) [61,62], a modified SHIM replacing “sexual intercourse” with “masturbation” in questions 3-5 [63]; the Erection Hardness Score (EHS) [64,65]; the Male Sexual Health Questionnaire–Ejaculatory Dysfunction–Short Form (MSHQ-EjD-SF) [66,67]; and a modified version of the first question of the MSHQ-EjD-SF, where “sexual intercourse” was replaced with “masturbation” (self-developed modification). The self-estimated intravaginal ejaculation latency time [68] and the self-estimated ejaculation latency time during masturbation (self-developed question) were additionally assessed. The frequencies of sexual intercourse, masturbation, nocturnal erection, and feelings of sexual desire were evaluated using point scales similar to those used in the 2023 National Survey in Japan [69].

Furthermore, the Aging Males’ Symptoms Scale (AMS) score [70-72] was used as an indicator of physical and psychological changes associated with androgen decline. The National Institutes of Health Chronic Prostatitis Symptom Index (NIH-CPSI) [73] was used to evaluate chronic prostatitis symptoms. These measures were included to support exploration of potential infertility risk factors and hypothesis generation.

Published studies support the reliability/validity or relative validity of the BDHQ [55], PSQI-J [56], rMEQ [57], IPAQ [58], AUDIT-C [54], PSS [59], K6 [60], AMS [71], and NIH-CPSI [73] in Japanese populations. A Japanese version of the SHIM [62] and MSHQ-EjD-SF [67] were used. The EHS is a single-item measure, for which internal consistency is not applicable. Modified or self-developed items were treated as exploratory measures.

#### Physical Measurements

For group 2 participants, we conducted detailed physical function evaluations and physical measurements based on standardized procedures. The weight, BMI, body fat percentage, muscle mass, and segmental muscle mass of the limbs and trunk were measured using a multifrequency body composition analyzer (MC-780A, TANITA, Tokyo, Japan). The skeletal muscle mass index [74], fat mass index [75,76], phase angle [77,78], and extracellular water–to–total body water ratio [79] were also measured. These indicators were collected to evaluate the detailed body composition, including regional characteristics and cellular status, in addition to indicators such as BMI and body fat percentage commonly used in previous studies [80,81]. Measurements were performed with participants barefoot and wearing light clothing, with corrections made for the weight of the clothing.

Subsequently, muscle strength was evaluated as a physical indicator of androgen status and whole-body muscle strength. In addition to grip strength [82,83], a widely used representative indicator of whole-body muscle strength and physical function, back muscle strength [84] was also measured for a more comprehensive evaluation of muscle strength status, including that of the trunk. Digital grip strength (TAKEI 5401, SANKA Co, Ltd, Health Equipment Division, Tokyo, Japan) and back muscle strength meters (TAKEI 54021, SANKA Co, Ltd) were used. Grip strength was measured twice for each hand alternately in the standing position, and the maximum value was adopted [85]. Back muscle strength was measured twice, with the upper body inclined forward at 30° and the knees extended (but not locked), and the maximum value was adopted [86].

Anatomical circumferences were measured based on standardized procedures using a nonstretchable measuring tape (SECA 201, Hamburg, Germany). Specifically, the waist circumference was measured at the midpoint between the lower rib margin and the iliac crest [87]. The maximum circumferences of the hip [88], thigh [89], and calf [90] were also measured. These circumferences were collected exploratorily for a simple evaluation of the regional body shape and fat/muscle distribution, which could not be captured using whole-body composition indicators. For quality control, each site was measured twice, and if the difference was ≥1 cm, a third measurement was performed. Sexual function may be associated with complex physical factors, such as systemic metabolic status, muscle mass/strength, fluid distribution, and physical activity. Thus, data on body composition, muscle strength, and morphological indicators were comprehensively collected to evaluate the whole-body physical status multidimensionally.

#### Semen Analysis and Subjective Assessment

Following an ejaculatory abstinence period of 48 to <144 h (verified upon arrival) [91], semen was collected via masturbation in a designated room at the clinical research facility and analyzed in an onsite laboratory. The samples were liquefied in a 37°C incubator or at room temperature, with a maximum liquefaction time of 60 minutes. Incomplete liquefaction was defined as a thread length of ≥2 cm during pipetting, in which case mechanical agitation using an 18-gauge needle was performed. Semen volume was calculated by weighing the collection container, assuming a semen density of 1.0 g/mL.

Semen analysis was performed using an automated semen quality analysis system (LensHooke X3 PRO, Bonraybio, Taichung, Taiwan) [92]. Sperm concentration and total motility were measured in accordance with the 6th Edition of the *World Health Organization (WHO) Manual for Human Semen Analysis* [93,94]. The following specific kinematic parameters were also analyzed: categorization of sperm motility (fast progressively motile, slow progressively motile, nonprogressively motile, and immotile), average path velocity, straight line velocity, curvilinear velocity, amplitude of lateral head displacement, beat cross frequency, linearity, straightness, and wobble [95]. In addition, the percentage of normal morphology, pH, and round cell concentration were measured using an automated image recognition system. To evaluate sperm oxidative stress, the static oxidation-reduction potential was measured using a sperm oxidative stress measurement system (MiOXSYS, Caerus Biotech, Vilnius, Lithuania) [96]. Microscopic videos generated by the system were stored in native output format, and file metadata (eg, frame rate and resolution) were retained for subsequent image-based analyses.

The participants additionally completed a Subjective Assessment at Collection questionnaire immediately after semen collection. The questionnaire recorded the abstinence period, presence of spillage, time taken for collection on the study day, subjective intensity of orgasm on the study day (4-point Likert scale) [97], subjective semen volume compared to usual (modified question 3 of the MSHQ-SF), subjective time taken for collection compared to usual (self-developed question), and stress level during collection (10-point Likert scale, self-developed question). This was intended to aid in assessing the influence of psychological and situational factors on sample quality.

Residual samples after analysis were preserved as smears for Diff-Quik staining. Residual semen for downstream exploratory analyses was aliquoted according to the volume available after onsite testing and stored at −80°C before shipment to collaborating institutions or external laboratories. The smears are being morphologically evaluated based on Kruger’s strict criteria [98]. Depending on residual sample availability, frozen semen aliquots are being used for exploratory analyses of the sperm DFI; high DNA stainability (HDS) using SCSA [99-101]; trace and major elements and chemical speciation elements in semen and seminal plasma; and additional biomarkers, such as inhibin B and insulin-like 3 (INSL3).

#### Blood and Urine Assays

Group 2 participants provided early-morning urine and fasting blood samples. Fasting was defined as no breakfast on the study day, and the participants were instructed to finish dinner by approximately 9:00 P.M. on the day before the visit. Water intake was permitted. To account for diurnal fluctuations in analytes, such as testosterone [102] and zinc [103] levels, blood samples were collected between 8:00 and 11:00 A.M. To avoid potential alterations in parameters such as postejaculation prolactin, blood sampling was performed before semen collection [104]. A portion of the blood was processed into plasma or serum and stored frozen at −80°C; these samples were shipped to collaborating institutions or external laboratories for exploratory analysis.

Urine assays include measurements of exposure to environmental chemicals (phthalate esters, parabens, and pesticides), soy isoflavones, trace and major elements, chemical speciation of elements, creatinine, specific gravity, and urea nitrogen. Blood assays include measurements of serum and plasma gonadotropins and sex steroid profiles (follicle-stimulating hormone, luteinizing hormone, testosterone, free testosterone, dehydroepiandrosterone sulfate, sex hormone–binding globulin, dihydrotestosterone, estradiol, prolactin, anti-Müllerian hormone, inhibin B, and INSL3), metabolic markers (serum albumin, fasting blood glucose, and insulin), and selected exposure substances (per- and polyfluoroalkyl substances, trace and major elements, and chemical speciation of elements) in whole blood, serum, and plasma.

#### Definition of Outcome Measures

The primary outcome measure is the continuous DFI. The DFI refers to the proportion of sperm with fragmented or acid-denaturable DNA. In this study, the DFI is measured using SCSA after acid treatment and acridine orange staining with flow-cytometric analysis [12-14]. The primary analysis will assess whether predefined microscopic sperm image-/video-derived summary variables are associated with a continuous DFI, as a feasibility-oriented proof-of-concept evaluation of whether sperm microscopy data contain information relevant to sperm DNA integrity. Secondary and exploratory outcome measures include a categorical DFI based on literature-informed or study-specific exploratory thresholds, HDS, and general semen analysis parameters. Clinical, environmental, physical, and lifestyle variables will be examined as candidate correlates or confounders in secondary exploratory analyses [105].

### Statistical Analysis

#### Descriptive Statistics and Data Preprocessing

Continuous variables will be summarized as means (SDs) and/or medians (IQRs), depending on the distribution characteristics, and categorical variables will be summarized as frequencies (percentages) [106]. The DFI will be treated as a continuous variable. Categorization using literature-informed thresholds (eg, 15% and 27%) [107-110] will also be used in secondary exploratory analyses. In addition, if data characteristics permit, a study-specific candidate threshold will be explored using receiver operating characteristic (ROC) analysis against a prespecified binary semen quality classification based on the lower fifth percentile values for basic semen parameters described in the 6th Edition of the *WHO Manual for Human Semen Analysis* [94,111,112]. Any such data-driven threshold will be considered hypothesis generating only [113].

No formal between-group inferential comparison between groups 1 and 2 is a primary objective of this study. Any comparison of baseline characteristics between the two groups will therefore be descriptive/exploratory only. If participant overlap between groups is present, co-enrolled individuals will be counted only once in such cross-group summaries or in any pooled participant-level analysis requiring independence. Under the primary handling rule, the group 1 record will be retained and the corresponding group 2 record excluded, because group 1 had stricter eligibility criteria and a quality control purpose. Alternative handling rules, such as retaining the group 2 record or excluding co-enrolled participants entirely, may be examined as sensitivity analyses. This issue does not affect the main group-specific analyses, in which group 1 is used for DFI quality control and group 2 for exploratory association analyses.

All statistical tests will be two-sided and interpreted using a nominal significance level of *P*<.05. However, the secondary analyses are exploratory and involve multiple related comparisons and sensitivity analyses. Thus, emphasis will be placed on effect sizes, 95% CIs, and consistency of direction rather than on statistical significance alone, and no formal multiplicity adjustment is planned. As exposure indices (eg, environmental chemicals) may generally exhibit skewness, transformations (eg, logarithmic transformations) will be considered according to the distribution [114]. For urinary indices, a correction using urinary creatinine or specific gravity will be applied [115,116].

#### Quality Control Analysis

Data from group 1 will be used for quality control of the DFI measurement system. Specifically, using SCSA for DFI and HDS measurements, we will evaluate intraday, interday, and interfacility variability using quality control semen samples. Given that SCSA-derived DFI values can be influenced by the assay protocol, instrument settings, and gating strategy, this quality control step is required before the main association analyses [14,117,118]. After evaluating measurement validity, the flow cytometry gating settings will be fixed to ensure measurement reliability. Using data from group 1, we will confirm the distribution in young, healthy men; exclude outliers; and describe the reference value range.

#### Image Information Analysis

For the primary analysis, predefined microscopic sperm image-/video-derived summary variables will be assessed for association with the continuous DFI (primary outcome measure) as a preliminary proof-of-concept evaluation of whether image/video data contain information relevant to sperm DNA integrity. Associations with the continuous DFI will initially be assessed using correlation analyses and simple linear regression. When the DFI is categorized using prespecified literature-informed thresholds or a study-specific exploratory candidate threshold, simple logistic regression will be used as a secondary exploratory analysis. Any predictive modeling (including AI approaches) will be considered exploratory and will be specified in subsequent phases after database expansion. Exploratory image-DFI association analyses will be reported separately by group. If any additional pooled participant-level analysis across groups 1 and 2 is performed, the nonoverlapping dataset described earlier will be used.

#### Exploratory Analysis

For group 2 data, associations among the DFI, microscopic sperm image/video data, and clinical variables will be analyzed to identify candidate predictors and confounding factors for image-based analyses. Core analyses will prioritize standard semen parameters, questionnaire data, and physical measurements. The DFI will be analyzed primarily as a continuous outcome, with secondary analyses using thresholds [107-110]. Building on the reference values established in the quality control analysis, we may explore a study-specific DFI threshold against the WHO criteria [93] using ROC analysis. The area under the ROC curve and Youden index–derived cut points will be reported as hypothesis-generating results.

Candidate variables will first be examined using correlation analyses, group comparisons, or simple linear/logistic regression, as appropriate to variable type. Age and recruitment-related participant characteristics (eg, employment status, shift work, education, and income) will also be examined as candidate covariates or stratification factors to evaluate potential selection-related bias. The data of participants selecting “prefer not to answer” for education or income will be retained in descriptive analyses, and sensitivity analyses will compare alternative handling of these responses (eg, separate-category vs exclusion approaches). Given that a population-based sampling frame is not available, these analyses are intended to assess robustness rather than fully remove selection bias. Multivariable linear or logistic regression models will be considered only when data completeness and the number of informative observations is adequate. To reduce overfitting, the number of predictors simultaneously included will be restricted, with priority given to variables selected based on prior knowledge and univariable results. Multicollinearity will be assessed using the variance inflation factor, with values >3 considered to indicate potential concern. In such cases, we will preferentially retain representative variables or use dimension reduction, clustering, or mixed-exposure approaches as exploratory analyses.

#### Missing Data and Sensitivity Analysis

Participants with missing data for primary outcome measure analysis (DFI or images) will be excluded from the primary analysis, and the reasons for missingness will be described. Considering that the status of questionnaires or samples, such as self-reports of spilling all or part of the sample during collection [119] or recent infectious diseases (eg, a cold, influenza, or COVID-19) with or without a high fever (≥38°C) within the past 3 months [120,121], may affect the results, sensitivity analyses excluding such participants will be conducted, as necessary. If azoospermic samples are observed, sensitivity analyses will be performed both including and excluding them because their underlying etiologies cannot be fully characterized in this non-clinic-based setting [122].

### Ethical Considerations

The study protocol underwent central ethical review and was approved by the Institutional Review Board (IRB) of the Osaka Pharmacology Clinical Research Hospital (IRB approval #R01114a; committee review date: December 18, 2025; notification date: December 19, 2025) in accordance with the Ethical Guidelines for Medical and Biological Research Involving Human Subjects in Japan. AIST, which leads protocol development and overall study governance, does not accept reliance on another institution’s review. Hence, independent approval was additionally obtained from the IRB of AIST (IRB approval #Life 2025-0618; review completion date: January 6, 2026; notification date: January 7, 2026). Based on the central review, study implementation or institutional approval was also obtained from the University of Tsukuba Hospital (approval #R07-275), the Tsukuba Gakuen Hospital (approval #25-23), the International University of Health and Welfare (approval #25-MB-18), the Nagoya University Graduate School of Medicine (approval #2025-0536; commission #2025-184), and the National Institute for Environmental Studies (approval #2025-024).

The study will be conducted in accordance with the Declaration of Helsinki and Ethical Guidelines for Medical and Biological Research Involving Human Subjects in Japan. Written informed consent was obtained from all participants after a full explanation of the study objectives, procedures, potential benefits, and risks. Questionnaire data and biospecimens were deidentified using a study identification code. The linkage key is retained only at the Osaka Pharmacology Clinical Research Hospital, and collaborating institutions and external laboratories receive coded data/samples only. Sensitive sexual and reproductive information was collected primarily through self-administered questionnaires, where participants were allowed to select a “prefer not to answer” option to ensure their autonomy and minimize psychological distress, consistent with a recent study [123]. The participants received reimbursement for study-related expenses to reduce the burden of transportation and time commitment rather than as an incentive for participation: JPY 10,000 (~US $63) for group 1 and JPY 15,000 (~US $94) for group 2 (JPY 5000, ~US $31, on the explanation day and JPY 10,000, ~US $63, on the study day). Individual research results, including incidental findings, are not routinely returned because the assays were conducted for research purposes and may not be clinically interpretable at the individual level. This policy was explained during the informed consent process.

## Results

Participant recruitment began on January 18, 2026, and recruitment and sample/data collection were completed on February 21, 2026. Final enrollment yielded 25 group 1 records and 103 group 2 records. In addition, 4 individuals contributed records to both groups; thus, the study comprises 124 unique participants overall. For any cross-group descriptive comparison or pooled participant-level analysis requiring independence, these co-enrolled cases will be handled using the prespecified de-duplication rule described in the *Statistical Analysis* section. The last participant visit occurred on February 21, 2026. Biological samples will be sequentially analyzed after collection, and exploratory assays are planned to continue throughout the study period until March 2028. The findings of this study will be disseminated through domestic and international academic conferences and journal publications. After completion of biospecimen assays, the primary association analysis (between sperm image/video data and the DFI) and exploratory analyses of secondary outcome measures will be conducted, with primary analyses planned to be completed by March 2028.

## Discussion

### Summary

The principal contribution of this initial phase of N-SEED is the establishment of a standardized methodology for constructing a prospective multimodal database that links sperm microscopy image/video data with the DFI and a wide range of environmental, physiological, and lifestyle covariates. Compared to much of the existing image analysis literature on male infertility, this protocol incorporates environmental chemicals, endocrine profiles, and lifestyle-related measures, alongside semen microscopy [18-20]. In this initial phase, the resulting resource is intended to support feasibility-oriented association analyses, confounder assessment, and hypothesis generation. It is not intended to provide a clinically deployable AI tool or definitive evidence regarding predictive performance.

### Comparison With Prior Work

Most current AI applications in male infertility have focused on relatively narrow tasks, such as sperm morphology, motility, sperm retrieval, or in vitro fertilization-related prediction. Recent reviews consistently note methodological heterogeneity, limited multimodal integration, and insufficient multicenter external validation as major barriers to clinical implementation [18-20]. More broadly, the literature on semen quality has also been affected by heterogeneous source populations, retrospective designs, and differences in laboratory methods, which complicate interpretation across studies [2,4].

Overall, these prior studies indicate two relevant gaps. First, datasets directly linking standardized sperm microscopy image/video data with a functional marker of sperm DNA integrity, such as the DFI, are limited [18-20]. Second, environmental, endocrine, physical, and lifestyle variables that may confound or contextualize image-based associations are often incompletely represented in existing datasets [18-20].

The protocol is specifically designed to address these methodological gaps by prospectively collecting standardized sperm microscopy image/video data, together with the SCSA-based DFI and broader covariates in apparently healthy community volunteers. By recruiting outside infertility clinics, the study attempts to reduce, although not eliminate, the disease-related selection structure that characterizes many retrospective clinic-based datasets. Accordingly, the immediate contribution of this initial phase is methodological: to establish standardized acquisition procedures, characterize data completeness and measurement variability, and support feasibility-oriented analyses of associations between image-/video-derived variables and the DFI. Questions of model development, threshold validation, and external generalizability remain for later expanded phases.

### Strengths and Limitations

The strength of this study lies in the establishment of two cohorts according to specific objectives. Group 1 was recruited to secure reliable reference measurements of the DFI through rigorous quality control and by minimizing major confounding factors. Group 2, which included participants from diverse backgrounds, was recruited to explore realistic risk factors. Another strength is the quantification of subjective ejaculation sensations and collection conditions to consider psychological and situational factors.

However, the study also has some limitations. The study has a cross-sectional design, which means that separate longitudinal studies are necessary to assess temporality and to validate any future image-based markers against fertility outcomes. In addition, single-point urine and blood samples may not fully capture long-term exposure trends. Another inherent limitation is the potential selection bias introduced due to volunteer panel recruitment and operational requirement for morning fasting visits (and two visits for group 2), which may preferentially include men with greater scheduling flexibility. Similar patterns have been reported in healthy-volunteer research, where participants can be disproportionately students or individuals with limited economic independence in some contexts [96,97]. Accordingly, this initial phase should be interpreted as a feasibility-oriented volunteer sample for association analyses and hypothesis generation rather than a population-representative prevalence study. Residual bias may remain because some socioeconomic variables permit nonresponse.

Moreover, the initial phase sample size was determined pragmatically to establish standardized data collection rather than to detect a prespecified small effect size. Thus, null or unstable findings, especially in subgroup or multivariable analyses, will require cautious interpretation and should primarily inform future sample size calculations and variable prioritization. Any study-specific DFI threshold derived from the dataset will also require validation in an independent dataset before it can be interpreted as a clinically useful decision threshold. Concurrently, the pragmatic scale of this initial phase and the centralized single-country collection design mean that this cohort should be regarded as a high-fidelity pilot resource rather than a definitive etiologic or model validation dataset. Although it may identify candidate image-DFI relationships and relevant confounders, it cannot establish small exposure effects, definitive DFI thresholds, or broad algorithmic generalizability without later expansion and independent external validation [19].

However, the primary purpose of this study is to verify feasibility and conduct proof-of-concept association analyses, thereby serving as a foundation for future large-scale research. This protocol does not describe a tool ready for immediate clinical implementation and does not provide individual AI-derived fertility scores to participants. Any future implementation would require careful ethical oversight, appropriate counseling, and external validation.

### Conclusion

This protocol describes the initial phase of the N-SEED initiative, which establishes a standardized and scalable framework integrating prospectively collected sperm microscopy image/video data, the DFI, and extensive environmental and physiological covariates in healthy adult men. The resulting dataset will enable association analyses to evaluate feasibility and generate hypotheses. It will also inform subsequent phases, including expansion of participant numbers, refinement of image acquisition procedures, and stepwise development and external validation of image-based analytical approaches (including AI/ML) against clinically relevant and, ideally, longitudinal fertility outcomes. The database structure is designed for long-term maintenance and iterative updates beyond the 2028 study end date, contingent on governance and resources. Thus, the immediate deliverable of the initial phase is a standardized, quality-controlled multimodal resource to support later hypothesis testing, model development, and external validation, rather than a finalized clinical AI tool.

## Data Availability

Data sharing is not applicable to this protocol paper. The dataset generated by the Nippon Semen and Environmental Exposure Database initiative will be stored in a controlled-access manner, and secondary use will be considered upon reasonable request and subject to ethical approval and appropriate data-sharing agreements.

## Abbreviations

AI: artificial intelligence
AIST: National Institute of Advanced Industrial Science and Technology
AMS: Aging Males’ Symptoms Scale
AUDIT-C: Alcohol Use Disorders Identification Test–Consumption
BDHQ: Brief-type Self-Administered Diet History Questionnaire
DFI: DNA fragmentation index
EHS: Erection Hardness Score
HDS: high DNA stainability
INSL3: insulin-like 3
IPAQ: International Physical Activity Questionnaire
IRB: Institutional Review Board
K6: Kessler Psychological Distress Scale
ML: machine learning
MSHQ-EjD-SF: Male Sexual Health Questionnaire–Ejaculatory Dysfunction–Short Form
NIH-CPSI: National Institutes of Health Chronic Prostatitis Symptom Index
N-SEED: Nippon Semen and Environmental Exposure Database
PSQI-J: Japanese version of the Pittsburgh Sleep Quality Index
PSS: Perceived Stress Scale
rMEQ: reduced Morningness-Eveningness Questionnaire
ROC: receiver operating characteristic
SCSA: sperm chromatin structure assay
SHIM: Sexual Health Inventory for Men
UMIN-CTR: UMIN Clinical Trials Registry
